# Predicting isoniazid resistance in *Mycobacterium tuberculosis* complex in New York State using whole-genome sequencing

**DOI:** 10.1128/jcm.01609-25

**Published:** 2026-03-18

**Authors:** Kruthikaben Patel, Joseph Shea, Pascal Lapierre, Tanya A. Halse, Donna Kohlerschmidt, Michelle Dickinson, Vincent Escuyer, Kimberlee A. Musser

**Affiliations:** 1 Wadsworth Center, New York State Department of Health 1094, Albany, New York, USA; The University of North Carolina at Chapel Hill School of Medicine, Chapel Hill, North Carolina, USA

**Keywords:** tuberculosis, isoniazid, drug resistance, whole-genome sequencing, *katG*, inhA

## Abstract

Isoniazid (INH) is a critical antibiotic used worldwide for the treatment and prophylaxis of tuberculosis. Drug resistance (DR) to INH is the single most common type of DR, mediated by multiple genes/loci, including *katG, inhA, mabA, mabA-inhA,* and the *oxyR-ahpC* intergenic region. Over the course of 6 years, we performed a two-phase study of 3,696 *Mycobacterium tuberculosis* complex (MTBC) strains, aiming to determine the molecular basis of INH resistance and assess whole-genome sequencing (WGS) for predicting resistance. In phase 1, we performed a side-by-side study, including 1,767 strains with paired phenotypic drug susceptibility testing (DST) and genotypic DST. We found WGS capable of accurately predicting INH resistance with a sensitivity of 90.3% and a specificity of 99.8%. The negative predictive value of WGS for INH susceptibility was 98.8%. Based on these findings, we developed a molecular testing algorithm where phenotypic DST (pDST) was reduced and applied this new testing algorithm in phase 2 to 1,929 MTBC strains, resulting in streamlined testing, reduced cost, and decreased turnaround time (TAT). The prevalence of INH resistance among MTBC strains in New York was found to be 10.2%. Of the 3,696 isolates tested, 337 were predicted INH-resistant by WGS. Of 41 additional strains exhibiting phenotypic INH resistance, 38 were found to have mutations in genes known to be associated with INH resistance. This study demonstrates the utility of WGS as a molecular tool for predicting INH DR and shows that the vast majority of INH resistance in MTBC has a molecular basis in known resistance loci.

**IMPORTANCE:**

Isoniazid (INH) is one of the two most critical antibiotics used as part of standard treatment of tuberculosis and is also used as preventative therapy for contacts of tuberculosis patients, despite having a higher rate of drug resistance than all other antibiotics used in standard therapy. Furthermore, isoniazid resistance typically precedes rifampin resistance in the development of multidrug-resistant TB. As such, the reliable detection of INH resistance is crucial for case management and to limit the acquisition of additional drug resistance. The present study describes a whole-genome sequencing approach to predicting INH resistance from clinical isolates and models how this technology can be used within a reduced phenotypic drug susceptibility testing algorithm to limit duplicate testing, saving resources and time while maintaining the sensitivity of resistance detection.

## INTRODUCTION

Tuberculosis (TB) is one of the most prevalent infectious diseases caused by *Mycobacterium tuberculosis* complex (MTBC) bacteria. According to the World Health Organization (WHO), in 2023, an estimated 10.8 million people fell ill with TB worldwide and caused an estimated 1.25 million deaths in 2023, despite being a preventable and curable disease. TB continues to be the world’s leading cause of death from a single infectious agent, especially in people with acquired immune deficiency syndrome, and a major contributor to the growing number of antimicrobial-resistant infections globally (1–5). Multidrug-resistant TB (MDR-TB) is a form of TB caused by bacteria that are resistant to isoniazid (INH) and rifampin (RIF), the two most effective first-line TB drugs (6). According to the WHO guidelines, detection of MDR-TB requires bacteriological confirmation of TB and testing for drug resistance (DR) using rapid molecular tests, culture methods, or sequencing technologies (7).

INH is one of the critical components of chemotherapy, used worldwide for both treatment and prophylaxis of TB. INH resistance often precedes RIF resistance in the development of MDR-TB (8). Therefore, rapid and accurate diagnosis of INH-resistant TB is essential to limit the spread of INH-resistant TB and reduce the likelihood of further acquisition of DR. Timely identification of MDR-TB cases will improve utilization of appropriate drug regimen treatments in patients and reduce the transmission of MDR-TB, resulting in better clinical outcomes (9). Mutations in several genes and intergenic regions, including *katG*, *inhA*, *ndh*, *mabA (fabG1), mabA-inhA (fabG1-inhA),* and *oxyR-ahpC,* have been associated with INH DR (10–15). The *katG* locus encodes the catalase/peroxidase enzyme that is essential for INH activity as it activates the pro-drug INH to an active drug (16). Therefore, the presence of any mutation altering the activity of *katG* will prevent the activation of the pro-drug, resulting in resistance. Active INH disrupts the mycolic acid biosynthesis by inhibiting the product of *inhA*, the NADH-dependent enoyl acyl carrier protein (ACP) reductase enzyme (17). Mutations in the promoter region cause the overexpression of INH’s target, NADH-dependent enoyl-ACP, resulting in an MIC increase (18).

Culture-based phenotypic drug susceptibility testing (DST) methods are still used frequently and remain the method of reference for resistance profiling. However, these tests are slow and rely on mycobacterial growth in the presence of a critical drug concentration to distinguish between resistant and susceptible phenotypes based on epidemiological breakpoints (19). Phenotypic DST (pDST) is labor-intensive, requiring specialized infrastructure and highly trained staff. Molecular methods to predict drug resistance can overcome some of the limitations of culture-based testing, particularly by providing results at reduced turnaround time (TAT).

Molecular methods used to detect INH DR historically have included line probe assays (LPA), Sanger sequencing of resistance-associated genes, and pyrosequencing (20–22). In 2016, the WHO approved the use of two LPAs: Hain GenoType MTBDR*plus* version 2 (Hain Lifescience, Nehren, Germany) and Nipro NTM + MDRTB detection kit 2 (Nipro, Tokyo, Japan). While these molecular assays can detect INH DR at greatly decreased TAT, pDST is more sensitive at detecting resistance due to rare variants and resistance mechanisms that are not captured by these molecular tests (23). Newer methods, such as next-generation sequencing (NGS), have increased the sensitivity of DR detection compared to previous molecular assays by increasing the number of resistance genes targeted and are important to expedite implementation of appropriate therapy and impact patients' outcomes. NGS includes both targeted NGS (tNGS) and whole-genome sequencing (WGS). tNGS assays are now endorsed by the WHO and include multiple commercially available tests, as well as laboratory-developed assays (24, 25).

Whole genome sequencing (WGS) is a powerful diagnostic tool, as it determines the complete DNA sequence of an organism’s genome but usually requires a cultured isolate. Unlike other molecular assays, WGS is capable of screening both known DR-associated genes and other less well-characterized loci in the MTBC genome. Therefore, a comprehensive list of mutations in target genes that confer DR can be generated, resulting in a reliable prediction of the drug susceptibility profile of a particular isolate, within a quicker timeframe.

The aims of this study were to determine the capacity of WGS to predict INH resistance and susceptibility of clinical MTBC isolates in New York State (NYS), and to evaluate a testing algorithm with reduced pDST. In the first phase of this study, paired phenotypic and genotypic data were compared to determine the molecular basis of resistance in strains exhibiting phenotypic INH resistance. These data were used to inform the construction of a testing algorithm with WGS as the primary method of susceptibility profiling and pDST as a reflex method for non-pan-susceptible strains, which was implemented for the second phase of this study. We found WGS capable of predicting INH resistance and susceptibility with high sensitivity and specificity, which led us to use WGS as our primary method of drug susceptibility testing. Strains with rare and novel mutations continue to have phenotypic testing performed to contribute to the growing knowledge base of INH resistance mechanisms and mutations and to improve the predictive power of molecular assays.

## MATERIALS AND METHODS

### Clinical isolates

A total of 3,696 MTBC strains were included in this study, consisting of one isolate from every culture-positive case in NYS over a period of 6 years between January 2016 and February 2022. All samples were received as isolates or cultured in-house from clinical specimens in the biosafety level 3 Mycobacteriology Laboratory at the Wadsworth Center, New York State Department of Health. An in-house developed semiquantitative real-time PCR assay targeting the multi-copy IS6110 and single copy region external to RD9 (ExtRD9) was used to confirm the presence of MTBC DNA in all the specimens and isolates that were received (22).

### DNA extraction

DNA extraction was performed on heat-inactivated (80°C for 1 h) 1 mL liquid culture aliquots of clinical isolates using a modified version of the InstaGene/FastPrep (IG/FP) method (26). Isolates cultured on solid media were suspended in 1 mL of 7H9 broth prior to heat inactivation. Specific modifications include the volume of IG added to the pellets was changed to be 130–200 µL based on the size of the pellet (130 uL for small <1 mm, 160 uL for medium 1–3mm, and 200 uL for large >3 mm) to increase DNA yield, and the 56°C incubation was reduced from 30 to 10 min.

### Phenotypic drug susceptibility testing

Phenotypic DST was performed using the liquid culture MGIT 960 system (BACTEC Mycobacterial Growth Indicator Tube [MGIT] 960 SIRE package insert; Becton, Dickinson) (0.1 and 0.4 µg/mL) and solid 7H10 agar proportion method (0.2 and 1.0 µg/mL) according to the Clinical and Laboratory Standards Institute’s recommendations (27). Prior to DST being set up, isolates were sub-cultured in MGIT medium. Strains exhibiting resistance at any of the above concentrations (0.1 µg/mL or higher) were considered INH resistant for sensitivity calculations. MGIT and agar proportion results were used for the concordance analysis in Table 2. MIC testing on a subset of isolates was performed by broth microdilution using two MIC plates: one commercially available Sensititre MYCOTB plate (ThermoFisher) and one custom plate (ThermoFisher) to further characterize mutations associated with INH resistance, but not used to determine or report INH resistance. The range of concentrations tested for INH was 0.03 to 4.0 µg/mL on the MYCOTB plate and 0.025 to 12.8 µg/mL on the custom plates. MIC testing was performed in triplicate, with plates being read by one analyst.

### Whole-genome sequencing

Paired-end sequencing was performed on the Illumina MiSeq and NextSeq platforms following Nextera Flex and Illumina DNA Prep library preparation, respectively. Sequencing runs were either all MTBC isolates or MTBC isolates with other bacterial, viral, and/or parasitic samples as previously described, but with improvements in cost and turnaround time described in Dickinson et al. (26, 28). WGS reporting includes an interpretation of resistant, susceptible, or unknown for each antimicrobial. For both reports of resistant and unknown, specific mutations are included in the report, including the gene/region, position, and nucleotide or amino acid change. For reports of unknowns, it is specified that no high-confidence mutations were detected.

### Bioinformatic analysis

Sequence analysis was performed using the Wadsworth Center TB WGS bioinformatics pipeline as previously described to detect an internally curated list of high confidence (HC) genomic insertions and deletions and single nucleotide polymorphisms (SNPs) for drug resistance profiling (26). Each HC mutation was individually validated as being associated with drug resistance, being found in multiple strains with paired phenotypic DST data exhibiting consistent results. Literature reports, particularly from well-curated databases with complete genetic information, were used to aid in the classification of less frequent variants; however, they were insufficient on their own to determine mutations as HC. Furthermore, all mutations in INH resistance-associated genes and gene regions were screened to identify rare and novel resistance mutations. Major lineage identification was based on the presence or absence of lineage-defining SNPs, with nomenclature according to Gagneux & Small and Feuerriegel et al. (29, 30).

## RESULTS

Universal WGS was performed on one isolate from every TB case in NYS with an available MTBC isolate over a 6-year period (*n* = 3,696). In the first phase of this study (January 2016–September 2018), side-by-side pDST (MGIT and agar proportion) and genotypic testing (WGS) were performed for all samples (*n* = 1,767). In the second phase (October 2018–February 2022), a tiered testing algorithm was implemented (*n* = 1,929) where phenotypic DST was limited to samples with known DR mutations (HC mutations) and mutations of unknown significance, to reduce the testing burden while still collecting phenotypic DST data to link with rare mutations and mutations of unknown significance (31). The current cost for WGS of a bacterial isolate on the NextSeq platform is $78.39 USD, a cost that has decreased over time from over $200 USD per sample when this testing was first implemented in our laboratory.

### Study phase 1

WGS and pDST were performed in parallel as soon as material was available; WGS was able to be done directly on samples received as isolates, while pDST required an initial culture stage to yield enough material for setup. TAT of WGS was an average of 8 days faster than MGIT DST and an average of 49 days faster than agar proportion DST. WGS showed high levels of concordance with phenotypic DST (90.3% sensitivity and 99.8% specificity, Tables 1 and 2). Among the 1,767 isolates, 169 were identified as resistant and 1,598 as susceptible by WGS. Among the isolates with no high-confidence mutations detected by WGS, 14 isolates exhibited phenotypic DR. Of these, 13 harbored one or more mutations in genes associated with INH resistance (10 *katG*, 1 *katG + oxyR-*ahpC, 1 *inhA,* and 1 *oxyR-ahpC + furA-*katG). Of the 169 isolates predicted resistant by WGS, phenotypic DST confirmed 130 as resistant and reported two as susceptible (*mabA-inhA*: T-8A and *katG*: insertion). The remaining 37 had phenotypic testing performed at another laboratory, and data were not available to us; however, all 37 had well-known mutations in *katG* and *mabA-inhA* intergenic region known to confer INH resistance (32, 33). Among the INH-resistant isolates (*n* = 169), 157 had a single HC mutation, and 12 had two HC mutations (Table 3).

**TABLE 1:** Summary statistics of WGS-determined high-confidence mutations and pDST during phase 1^*b*^

		Phenotypic DST^*a*^			
		R	S	ND	Total
**WGS**	R	130	2	37	169
	S	14	1,135	449	1,598
	Total	144	1,137	486	1,767

^*a*^ INH 0.1 µg/mL was used to determine resistance.

^*b*^ R, resistant; S, susceptible; ND, not determined; no result available.

**TABLE 2:** Summary statistics of WGS high-confidence mutations with pDST and INH resistance prevalence during phase 1^*a*^

Performance characteristics	Phase 1
Sensitivity % (n)	90.3 (130/144)
Specificity % (n)	99.8 (1,135/1,137)
Positive (resistance) predictive value % (n)	98.5 (130/132)
Negative (susceptibility) predictive value % (n)	98.8 (1,135/1,149)
Accuracy % (n)	98.7 (1,265/1,281)

^*a*^ Phase 1: 1,767 total isolates, 486 samples did not have pDST data available.

**TABLE 3:** Frequency of HC mutations in 169 INH-resistant isolates during phase 1

DST pattern	N	Amino acid change				
		*katG*	*mabA-inhA*	*mabA*	*inhA*	*oxyR-ahpC*
INH resistant	64	Ser315Thr				
	34		C-15T			
	8			Leu203Leu		
	3		G-17T			
	3	Ser315Asn				
	3	Deletion				
	3	Ser315Thr		Leu203Leu		
	3	Ser315Thr	C-15T			
	2	Gly121Ser	C-15T			
	2				Ser94Ala	
	1	Insertion				
	1	Ser315Thr	T-8C			
	1	Gln525Pro	C-15T			
	1		C-15T		Ser94Ala	
	1	Deletion				C-81T
INH susceptible	1	Insertion				
	1		T-8A			
INH not determined	26	Ser315Thr				
	8		C-15T			
	3	Deletion				
Total	169					

### Study phase 2

A WGS prediction scheme categorizing MTBC strains as INH-resistant (HC mutations), unknown, or susceptible (R/U/S) based on the mutation profile was implemented and strains predicted to be susceptible had no routine phenotypic DST performed. Phenotypic testing is still being performed if other resistance is detected and in cases of suspected relapse or recurrence, as needed. The prediction category of unknown allows for targeted phenotypic testing to be performed on a subset of samples that are more likely to be INH resistant. Mutations of unknown significance that were found to have a resistant phenotype were most commonly found in *katG*. During this phase of the study, the list of HC mutations used to predict INH resistance was updated to include *inhA* Ser94Ala, *katG* Gly121Asp, Trp191Gly, and Thr394Ala. Each of these mutations accumulated enough data to support their association with resistance during phase 1 of this study, each being found in more than three strains with a resistant phenotype and no other potential resistance mutations in genes associated with INH resistance.

Among the 1,929 isolates in phase 2, 168 were predicted as INH resistant, 1,651 as INH susceptible, and 111 as unknown by WGS. Of the 111 with mutations of unknown significance, 25 exhibited phenotypic resistance, 15 of which had mutations in the *katG* gene, six had mutations in *katG* and *oxyR-ahpC*, two had mutations in the *furA-katG* promoter region, and one had a mutation in *mabA*. Among the isolates predicted to be susceptible by WGS, two with no mutations in known resistance loci were reported to have an INH-resistant phenotype by ancillary laboratories and had phenotypic testing performed to confirm the result. Of the 168 predicted resistant by WGS, phenotypic DST confirmed 97 as resistant. The remaining 71 had no phenotypic DST data available; however, they had well-known mutations in *katG, inhA, mabA,* and the *mabA-inhA* promoter region, which are associated with INH resistance (32, 33). Among the INH-resistant isolates (*n* = 168), 163 had a single HC mutation, and five had two HC mutations (Table 4).

**TABLE 4:** Frequency of HC mutations in 168 INH-resistant isolates during phase 2

INH phenotype	n	Amino acid change			
		*katG*	*mabA-inhA*	*mabA*	*inhA*
Resistant	61	Ser315Thr			
	25		C-15T		
	4			Leu203Leu	
	2		T-8C		
	1	Ser315Asn			
	1	Deletion			
	1				Ser94Ala
	1	Ser315Thr		Leu203Leu	
	1		G-17T		Ser94Ala
Not determined	39	Ser315Thr			
	24		C-15T		
	3			Leu203Leu	
	2	Ser315Thr	C-15T		
	1				Ser94Ala
	1		T-8C		
	1	Ser315Thr		Leu203Leu	
Total	168				

### Combined data sets summary

**Fig 1:**
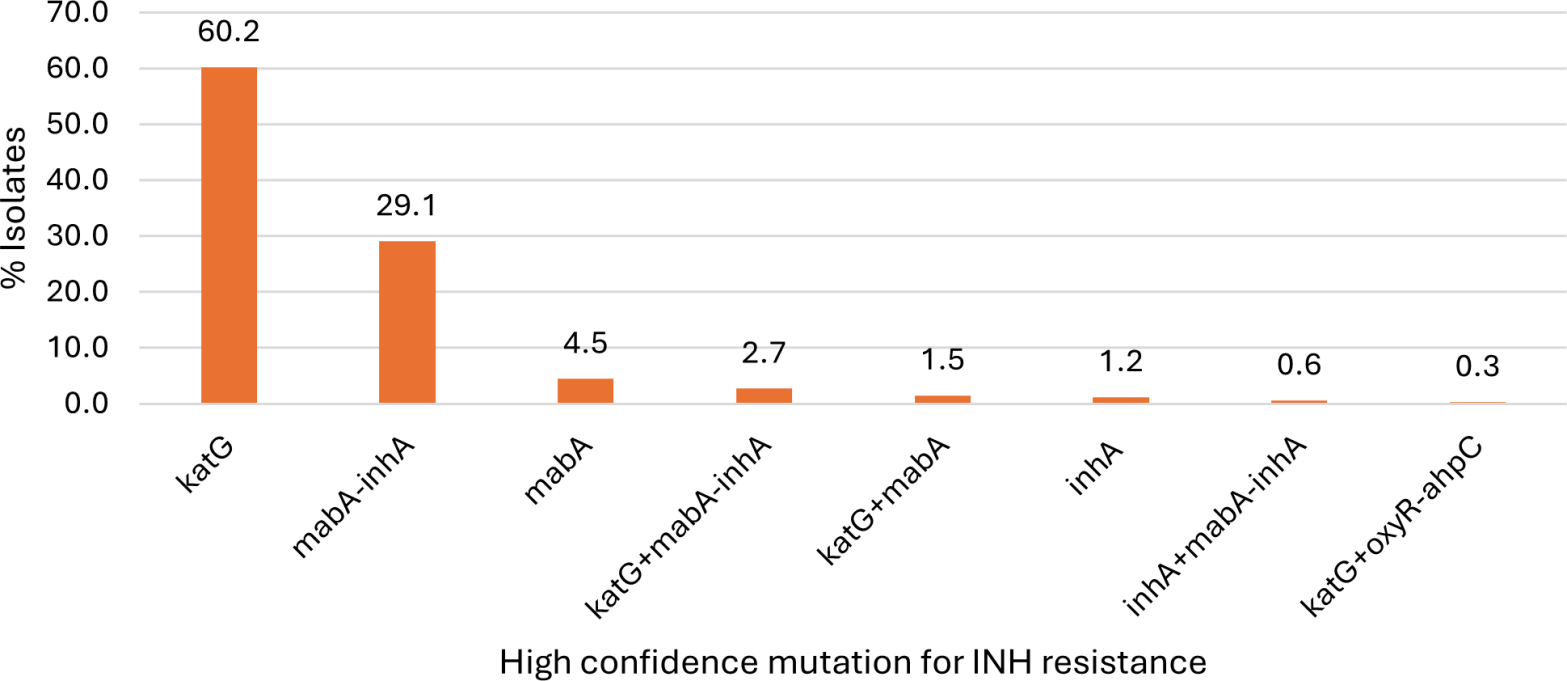
Proportion of resistance mutations in each gene/gene region in INH-resistant MTBC isolates.

Of a total of 3,696 isolates tested during phases 1 and 2, 337 were found to have HC mutation(s) in one or more genes associated with INH resistance (*katG*, *mabA-inhA*, *mabA*, *inhA*, and *oxyR-ahpC*) (Table 5). Of these, 312 (92.6%) isolates had a mutation in a single gene/locus, while 16 (4.7%) had mutations in multiple loci. The remaining nine (2.7%) had insertions or deletions in *katG*. Of the 337 INH-resistant isolates, 60.2% of HC mutations were found in *katG* (*n* = 203), followed by *mabA-inhA* (*n* = 98; 29.1%), *mabA* (*n* = 15; 4.4%), *inhA* (*n* = 4; 1.2%), *oxyR-ahpC* (*n* = 1; 0.3%) and the remaining 4.7% had HC mutations in two separate genes (Fig. 1). The predominant HC mutation was *katG* Ser315Thr, which was found in 190/337 (56.4%) of the strains predicted INH resistant by WGS. Other frequent HC mutations included *mabA-inhA* C-15T in 91 strains (27.0%) and *mabA* Leu203Leu (CTG to CTA) in 15 strains (4.5%). Among 268 total isolates found to have phenotypic INH resistance, 227 (84.7%) had HC mutations, and 39 (14.5%) had mutations of unknown significance in genes associated with INH resistance. In total, 266/268 (98.9%) strains with phenotypic INH resistance were found to have a possible molecular basis of resistance by WGS.

**TABLE 5:** WGS predictions and corresponding pDST results for INH in 3,696 MTBC isolates

	pDST resistant	pDST susceptible	pDST not determined	Total
WGS resistant	227	2	108	337
WGS susceptible	17	1,329	1,903	3,249
WGS unknown	24	78	8	110
Total	268	1,409	2,019	3,696

### Phenotypic drug susceptibility testing

**Fig 2:**
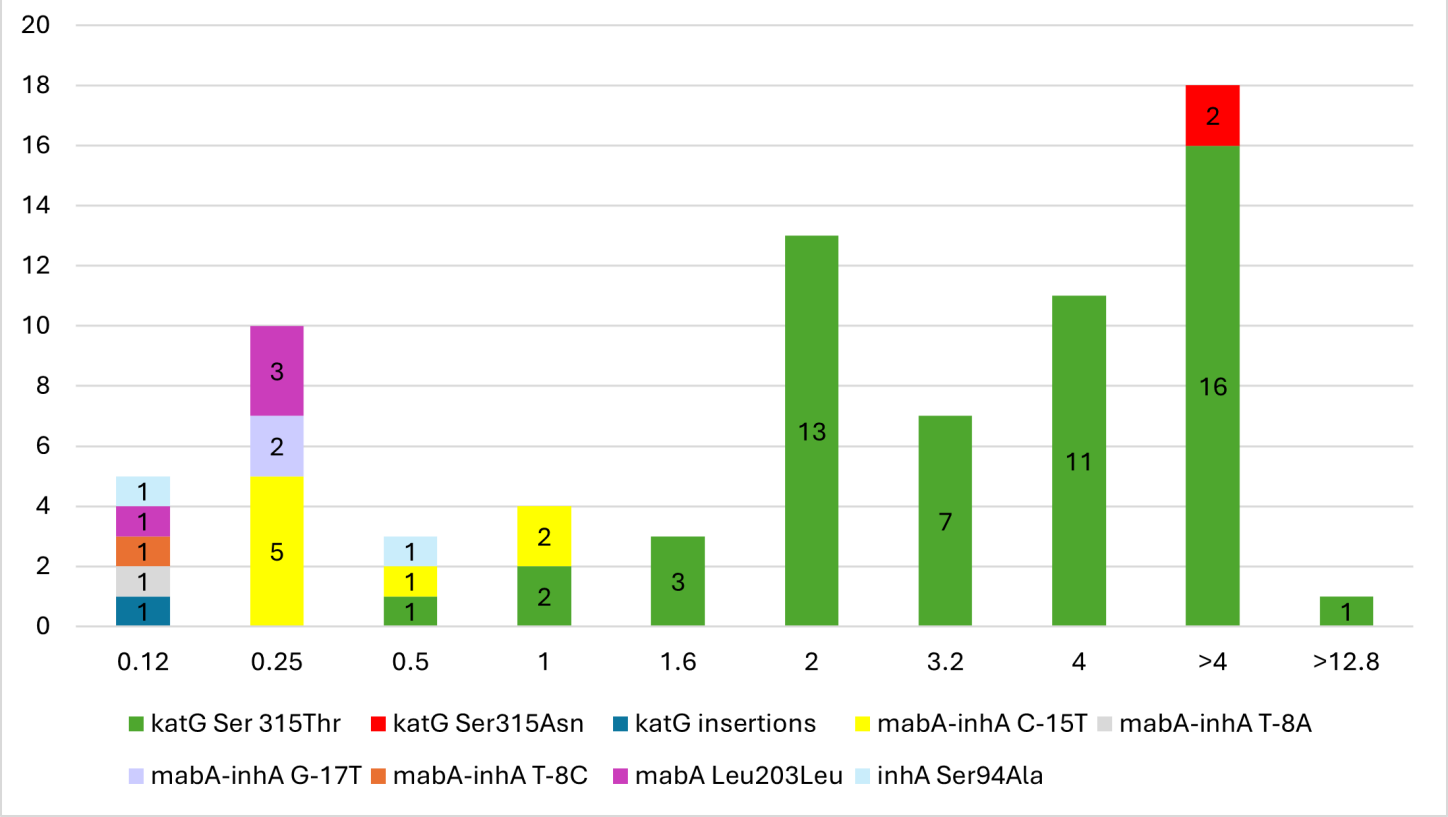
INH MIC distribution of HC mutations in MTBC isolates (*n* = 75).

DST data were available for 1,677 (45.4%) MTBC isolates based on MGIT DST. Of these, 268 (15.9%) were INH resistant, with 227 harboring HC mutations. The remaining 41 isolates exhibited phenotypic resistance, of which 39 harbored mutations of unknown significance in resistance-conferring genes (Table 6). MIC data were available only on a subset of the isolates included in this study. Of the 337 INH-resistant isolates, 75 (22.3%) had MIC data available, and the distribution of corresponding HC mutations with level of resistance is shown in Fig. 2. When tested by broth microdilution assay, the majority of the strains (50/75) were highly resistant to INH with MIC values over 2 µg/mL and had mutations in the *katG* gene codon 315. Of these, 30/50 had an MIC ≥4 µg/mL. Mutations in other genes explored in this study conferred MIC values ranging from 0.12 to 1 µg/mL. For example, the most common mutation in the *mabA-inhA* intergenic region, C-15T, had an MIC ranging from 0.25 to 1 µg/mL, and the less common mutations, such as T-8A and G-17T, had an MIC of 0.12 and 0.25 µg/mL, respectively. The silent mutation Leu203Leu in the *mabA* gene had an MIC of 0.12–0.25 µg/mL.

**TABLE 6:** Potential resistance mutations in isolates that were phenotypically INH-resistant (*n* = 39)

Resistance gene/gene region	Amino acid/nucleotide change	No. of isolates
*katG*	Ala93Val	1
	Ala144Val	1
	Ala162Thr	1
	Arg128Trp	1
	Arg249Gly	1
	Arg484Cys	1
	Arg595Gln	3
	Asp94Ala	1
	Gly121Asp	1
	Gly123Glu, Ala706Glu	1
	Leu427Pro	1
	Phe408Val	1
	Phe540Ser	1
	Ser315Gly	1
	Thr271Pro	1
	Thr344Pro, Gln352His	1
	Thr394Ala	2
	Trp191Gly	1
	Trp300Arg	1
	Tyr597Cys	1
*katG + oxyR-ahpC*	Ala109Thr + T-77A	1
	Gln127Pro, Lys157Glu + C-52A	1
	Gly299Ala + C-54T	1
	Met176Val + C-72T	1
	Trp191Gly + G-48A	1
	Trp191Arg + G-51A	1
	Val731Gly + C-52T	1
*furA-katG*	A-9C	2
*katG + ndh*	Val1Val (GTG to GTA) + Gly313Arg	1
*furA-katG + oxyR-ahpC*	A-10C + C-54T	1
*inhA*	Ile21Val	1
*ndh*	Arg268His Arg284Trp	1 1
	Insertion	1
*mabA*	Val221Val (GTC to GTA)	1

### INH resistance and mutation prevalence by major lineage

All four major lineages of *M. tuberculosis* (L1, L2, L3, and L4) were represented in our data set (14.4%, 22.3%, 8.4%, and 51.2% of strains, respectively). The distribution of strains with INH resistance-conferring mutations across these four lineages varied drastically, ranging from 19.3% in L1, 32.6% in L2, 3.9% in L3, and 43.9% in L4. INH resistance mutations were over-represented in L1 and L2 strains, given the prevalence of these lineages in this study (Table 7). We also found 131 strains to be other members of the MTBC (52 *Mycobacterium bovis*-BCG, 37 *Mycobacterium bovis*, 34 *Mycobacterium africanum*, 6 *Mycobacterium orygis*, 1 *Mycobacterium caprae*, and 1 strain of novel lineage La4) (34); none of these strains exhibited any INH resistance. Of the 52 *M. bovis*-BCG strains, 51 were isolated from patients with a history of BCG treatment for bladder cancer, and 1 was isolated from a pediatric patient after receiving BCG vaccine.

**TABLE 7:** Isoniazid resistance and mutation prevalence by major lineages

Lineage	No. of strains (%)	No. of INH-resistant strains by WGS (%)
L1	531 (14.4)	65 (19.3)
L2	526 (22.3)	110 (32.6)
L3	312 (8.4)	13 (3.9)
L4	1,892 (51.2)	148 (43.9)
*M. bovis* BCG	52 (0.01)	
*M. bovis*	37 (0.01)	
*M. africanum*	34 (0.009)	
*M. orygis*	6 (0.002)	
L7	1 (0.0003)	
La4	1 (0.0003)	
Mix of two lineages	1 (0.0003)	1 (0.003)
*M. caprae*	1 (0.0003)	
Total	3,696	337

## DISCUSSION

The last decade has seen tremendous advances in NGS technologies, including the use of WGS as part of the routine clinical diagnosis for infectious diseases. Since its implementation in our laboratory in January of 2016, clinical WGS for MTBC has improved surveillance and detection of INH resistance while providing insights into the molecular basis of INH-resistant strains in NYS. In this 6-year study, we analyzed a set of 3,696 MTBC isolates from unique patients to assess the frequency and type of INH resistance-conferring mutations determined by WGS and their association with phenotypic DST test results, including MIC testing. Data collected in the first phase of this study, reduced TAT compared to phenotypic testing, and a high negative predictive value of INH susceptibility were critical in guiding a testing algorithm change implemented for the second phase of this study. Additional data were collected to evaluate the relationship of strain lineage with INH resistance.

Past reports have shown that mechanisms of INH resistance are highly complex due to the involvement of several genes and intergenic regions (17). The present study focused on three genes and two regulatory regions associated with INH resistance mutations. These loci were screened for both high-confidence resistance mutations, internally validated with paired phenotypic and rare/novel variants. Although there is significant overlap between our internally validated HC mutations and those reported as being associated with INH resistance in the WHO catalog, we chose our smaller HC mutation list as it has been curated primarily using data collected in-house and is used to predict drug resistance in a clinical capacity (35). Resistance mutations were found predominantly in *katG* and *mabA-inhA*, with a smaller proportion in *mabA*, *inhA*, and *oxyR-ahpC*.

Mutations in *katG* confer INH resistance by blocking the pro-drug activation pathway. A variety of resistance-conferring mutations have been identified thus far in *katG*, though mutations in codon 315 are the most frequent and reportedly confer moderate to high levels of INH DR (36). In the current study, the prevalence of *katG* mutations in INH-resistant strains (60.2%) was consistent with previous reports, which have shown that mutations in the *katG* gene are the most prevalent, with 60%–67% of INH-resistant clinical TB isolates harboring mutations in this gene (18, 32, 37, 38). Among the INH-resistant isolates with *katG* codon 315 mutations, the most common nucleotide change, Serine to Threonine, occurred in 97.9% of the isolates. This is similar to a systematic review published in 2015, which reported that 93.4% of the phenotypic INH resistance was associated exclusively with a single Ser315Thr mutation in *katG* (32). Although resistance mutations in *katG* were found to occur most frequently at codon 315, we also found 31 INH-resistant strains to have other, less typical mutations in *katG*. These mutations were spread throughout the *katG* gene, not suggestive of a resistance-determining region as has been described for rifampin in *rpoB* and fluoroquinolones in *gyrA* and *gyrB*.

Mutations in the *mabA-inhA* intergenic region confer INH resistance by overexpression of the drug target, resulting in removal of the drug from the bacterial cell. Such mutations have been associated with a lower level of phenotypic DR compared to other mutations, most notably *katG* Ser315Thr (18). In the current study, 29.1% of the INH-resistant isolates had mutations in the *mabA-inhA* intergenic region, which is consistent with other studies that have reported 20%–42% of INH-resistant clinical MTBC isolates having mutations in this region (39–42). The most prevalent *mabA-inhA* intergenic region mutation is the C-15T mutation present in 92.2% of INH-resistant isolates with mutation in this intergenic region worldwide, which is similar to what was observed in this study (92.8%, 91 of 98) (32). Although the C-15T mutation is the dominant mutation in the *mabA-inhA* intergenic region, other resistance-associated mutations (7%) were observed.

A synonymous mutation in *mabA*, Leu203Leu (CTG to CTA), confers INH resistance by creating an alternative promoter for *inhA*, resulting in its overexpression (43). In most cases, silent mutations do not play a role in DR; however, the Leu203Leu mutation demonstrates that a silent mutation can be associated with DR depending on the specific gene and the location of the mutation. In the present study, 20 INH DR strains were found to have this *mabA* Leu203Leu mutation, occurring independently of other resistance mutations in 15 instances. The *inhA* gene is an essential gene implicated in INH resistance, though it was found not to harbor mutations as frequently as other resistance loci. Six of the seven *inhA* resistance mutations were Ser94Ala, the most reported resistance mutation in this gene (17).

Another locus evaluated was the *oxyR-ahpC* regulatory region. Mutations in this region of *katG*-defective INH-resistant MTBC strains have been described, which result in upregulation of AhpC, an alkyl hydroperoxidase, and thus compensate for the loss of catalase-peroxidase activity (44–47). However, mutations in the *oxyR-ahpC* regulatory region have also been identified in INH-susceptible MTBC isolates, suggesting that these mutations themselves may not confer INH resistance but may be selected for in strains which are INH resistant (48). All nine INH-resistant isolates found to have mutations in *oxyR-ahpC*, numbered in this study based on position relative to the start codon of *ahpC* (G-48A, G-51A, C-52A, C-52T, two C-54T, C-72T, T-77A, and C-81T), had mutations in *katG,* which could account for the phenotypic resistance. These mutations are believed to result in AhpC overexpression and partially restore the fitness cost due to reduced *katG* activity (49).

Combinations of mutations in *katG* and the *mabA-inhA* intergenic region are known to confer high-level resistance (50). In the current study, five isolates carrying the C-15T mutation also had the Ser315Thr mutation in the *katG* gene. An additional eight strains were found to have HC mutations in *katG* and another resistance gene, suggestive of sequential acquisition of resistance mutations resulting in higher-level INH resistance.

The MIC for *Mycobacterium tuberculosis* to INH varies significantly, with sensitive strains typically having MICs below 0.2 μg/mL, while isoniazid resistance is associated with higher MICs, ranging from above 2 μg/mL up to >10 µg/mL. These resistance levels are often linked to mutations in the *katG* or *inhA* genes, though the specific MIC values can overlap and are influenced by the particular mutation present (45, 50, 51). Our results are largely in agreement with the associations between known resistance-conferring mutations and the level of phenotypic resistance described in the literature. MICs for INH-resistant isolates with Ser315Thr mutation in *katG* (*n* = 54) were in the range of 0.5 to ≥12.8 µg/mL, and the MICs for isolates with amino acid replacements other than threonine at codon 315 of *katG* were ≥4 µg/mL (Fig. 2). Previous studies have shown that 61.7%–100% of the mutations at codon 315 in the *katG* gene, most commonly Ser315Thr, exhibit higher levels of INH resistance (36, 52, 53).

In our analysis, isolates with mutations in the *mabA-inhA* intergenic region (*n* = 12) had MIC values ranging from 0.12 to 1 µg/mL, corroborating the findings by Lempens et al., who reported that 50% of the isolates with C-15T mutation showed lower levels of INH resistance. The same study also reported that 5.8% of the isolates with C-15T mutation had higher MIC values ranging from 3.2 to 12.8 µg/mL. Higher level INH resistance in isolates carrying C-15T mutation can be explained if it coincides with a mutation in *katG*, especially Ser315Thr, or by mutations in other resistance loci (54). While WGS was found to predict INH resistance reliably, it is more challenging to predict the level of resistance or MIC from sequencing data. Broadly, *katG* mutations tend to result in higher levels of resistance, while mutations in *mabA-inhA*, *inhA*, and *mabA* typically correspond to lower-level resistance. Nevertheless, the same mutations in different strains, particularly those of different lineages, may have varying impacts on MIC. This is further complicated by rare mutations, especially in *katG*, which may not confer a higher level as reliably as Ser315Thr, and double mutations, which often result in much higher MIC than when present as solo mutations. Further studies, particularly with large-scale MIC testing, are needed to evaluate if predicting the level of resistance from sequencing data is feasible.

We provide further evidence that much of the resistance to INH in MTBC can be explained by mutations in known resistance loci. Of the 268 isolates with phenotypic INH resistance, 265 (98.9%) had mutations that could account for the phenotypic resistance. While most (227) of these had HC mutations (84.7%), 38 isolates had less well-characterized mutations and required phenotypic testing for final determinations of resistance. Five of the 38 isolates had *katG* mutations (one Gly121Asp, two Thr394Ala, and two Trp191Gly) that we confirmed to be associated with INH resistance and have now been included in our in-house pipeline as HC mutations. Of the remaining 33 isolates, 10 had *katG* mutations that are mentioned in the WHO catalog as mutations of uncertain significance, and two had *ndh* mutations of uncertain significance; however, recent studies have shown that *ndh* is not involved in INH resistance (35, 55). Targeted testing to identify only the most common resistance mutations, such as *katG* Ser315Thr and *inhA* C-15T, cannot fully replace conventional DST, and this study provides further evidence that less common mutations in *katG*, *inhA*, *mabA*, and *mabA-inhA* also account for INH resistance in clinical strains. Early detection of these less common INH resistance-associated mutations could decrease the chances of treatment failure and additional acquisition of DR.

Across both phases of this study, three isolates were found to have INH resistant phenotype despite not having mutations in known resistance loci. Additional samples initially discrepant were resolved with additional testing; most often, contamination resulted in errant phenotypic resistant results, which did not replicate when repeated on pure cultures. Of the three strains with discrepant results, each had no candidate mutations in INH resistance genes and was resistant by MGIT DST at 0.1 µg/mL. MIC testing was performed upon confirmation of the resistant phenotype result, and one strain was resolved to have a low MIC in the range of INH susceptible strains, suggesting the MGIT result may not be reliable. However, the other two strains had MIC at the low end of resistant strains (0.12 µg/mL). Notably, both strains were of the same genetic background, Lineage 1. Lineage effect on MIC has been more well-documented for other drugs, predominantly pyrazinamide, and INH resistance through an as-yet-unknown mechanism cannot be ruled out. In cases of discrepant results between molecular and conventional phenotypic testing, MIC testing can offer additional information and help make a determination about whether or not a strain is INH resistant. Furthermore, MIC testing is ideal when assessing rare and novel variants to determine if they play a role in resistance and to get the most complete information possible. Increased MIC testing on strains with atypical variants has been made possible by reducing the amount of conventional phenotypic testing, targeting our efforts to the strains and cases where additional testing offers the most benefit.

This study explored the prevalence of INH resistance-associated mutations in three genes and two regulatory gene regions reported to be associated with INH resistance in 3,696 clinical MTBC isolates. Based on the strong correlation observed between WGS and phenotypic DST in phase 1, a WGS prediction scheme categorizing the presence or absence of mutations as predictive of INH-resistant, unknown, or susceptible (R/U/S) phenotypes, and a tiered testing algorithm was implemented in phase 2. In phase 2, phenotyping testing was limited to samples with known resistance mutations and mutations of unknown significance. Some of the mutations of unknown significance were critical to detect instances of INH resistance, which would have otherwise gone undetected; this was primarily due to rare mutations in the *katG* gene. Our findings in this study highlight the importance of tracking mutations of unknown significance and linking them with phenotypic testing results to improve molecular predictions of INH resistance. This work will continue to contribute in the long term to the improvement of our WGS prediction algorithm for INH DR, though the accumulation of data for rare variants can be slow even with collections of thousands of strains. Larger international efforts, such as the WHO catalog, may overcome this with access to a more strain diversity and increased sample size than can be achieved at a regional level.

 WGS is a valuable tool for identifying genome-wide variants and has proven to be effective in detecting resistance to INH. Implementation of TB WGS has resulted in the rapid and accurate identification of INH resistance and multidrug resistance in MTBC isolates in NYS. In the context of a reduced pDST testing algorithm, WGS has decreased the amount of phenotypic testing required, reducing cost and duplicative testing while still reliably detecting INH-resistant tuberculosis. Over time, this work contributes to the efforts to characterize the molecular basis of INH resistance in tuberculosis and to improve the power of molecular assays at predicting drug resistance.
